# Effect of ginger and P6 acupressure on chemotherapy-induced nausea and vomiting: a randomized controlled study

**DOI:** 10.1590/1980-220X-REEUSP-2023-0104en

**Published:** 2024-03-04

**Authors:** Xiao Chenbing, Xia Huiling, Xing Qianqian, Wang Dan, Xie Guilan, Yang Ling, Xie Lingling, Qian Weiwei

**Affiliations:** 1 Xuzhou Medical University, School of Nursing Care, Xuzhou, China.; 2 Affiliated Hospital of Xuzhou Medical University, Department of Oncology, Xuzhou, China.; 3 Affiliated Hospital of Xuzhou Medical University, Department of Nursing Care, Xuzhou, China.

**Keywords:** Acupressure, Drug Therapy, Nausea, Ginger, Vomiting, Acupresión, Quimioterapia, Náusea, Jengibre, Vómitos, Acupressão, Tratamento Farmacológico, Náusea, Gengibre, Vômito

## Abstract

**Objective:**

To evaluate the effect of ginger with P6 acupressure in preventing and treating chemotherapy-induced nausea and vomiting (CINV) in cancer patients.

**Method:**

A total of 172 participants were randomly divided into the control, ginger, acupressure, and joint groups, who were hospitalized in the Affiliated Hospital of Xuzhou Medical University from February and September 2022. The baseline characteristics, nausea, vomiting, and retching, benefit finding, functional living index-emesis, treatment satisfaction, and adverse reaction, were used in data collection.

**Results:**

No significant difference was found in benefit finding and adverse reactions among the four groups (*P* > 0.05). Ginger significantly improved delayed CINV and function living index-nausea (*P* < 0.05) but had no significant effect on acute CINV, retching, and delayed vomiting, functional living index-emesis, and treatment satisfaction (*P*>0.05). Acute nausea and retching, delayed nausea, vomiting, and retching, functional living index-emesis, and treatment satisfaction were effectively improved in the acupressure and joint groups (*P* < 0.05).

**Conclusion:**

Ginger with P6 acupressure may contribute to improving CINV in patients undergoing chemotherapy.

## RESUMEN

**Objetivo:**

Evaluar el efecto del jengibre con acupresión P6 en la prevención y tratamiento de las náuseas y vómitos (nviq) inducidos por la quimioterapia en pacientes con cáncer.

**Método:**

Se dividió al azar A un total de 172 participantes en los grupos control, jengibre, acupresión y conjunto, que fueron hospitalizados en el Hospital afiliado de la universidad médica Xuzhou entre febrero y septiembre de 2022. En la recolección de datos se utilizaron las características basales: náuseas, vómitos y náuseas, hallazgo de beneficios, índice de vida funcional, satisfacción con el tratamiento y reacciones adversas.

**Resultados:**

No se encontró diferencia significativa en el hallazgo del beneficio y las reacciones adversas entre los cuatro grupos (P >0.05). El jengibre mejoró significativamente las nviq diferidas y el índice de vida funcional (nausea) de forma significativa (P < 0.05), pero no tuvo un efecto significativo en las nvi, las náuseas y el vómito diferidos, la emesis del índice de vida funcional y la satisfacción con el tratamiento (P >0.05). Las náuseas agudas y las arcadas, las náuseas, los vómitos y las arcadas tardías, la medida del índice de vida funcional y la satisfacción con el tratamiento mejoraron efectivamente en los grupos de acupresión y articulares (P < 0,05).

**Conclusión:**

El jengibre con acupresión P6 puede contribuir a mejorar las nviq en pacientes sometidos a quimioterapia.

## RESUMO

**Objetivo:**

Avaliar os efeitos da compressão do acuponto gengibre e Neiguan na prevenção e tratamento de náuseas e vômitos induzidos por quimioterapia em pacientes oncológicos.

**Métodos:**

Um total de 172 pacientes hospitalizados no Hospital Afiliado da Xuzhou Medical University de fevereiro a setembro de 2022 foram divididos aleatoriamente em grupo controle, grupo gengibre, grupo acuponto e grupo combinado. A recolha de dados incluiu principalmente dados basais, pontuação de náuseas, vómitos e vómitos, sensação de benefício da doença, índice de vida funcional, satisfação com o tratamento e efeitos adversos.

**Resultados:**

Não houve diferenças significativas no benefício da doença e efeitos adversos entre os quatro grupos (P >0,05). O gengibre melhorou significativamente o índice de vida funcional tardia e náusea (P < 0,05), mas não melhorou a NVI tardia e vómitos e vómitos retardados, o índice de vida funcional dos vómitos e a satisfação com o tratamento (P >0,05). Ambos os grupos de acuponto e combinação melhoraram náuseas agudas, vómitos, náuseas tardias, vómitos, vómitos, vómitos, índice de vida funcional e satisfação com o tratamento (P < 0,05).

**Conclusão:**

A acupuntura de gengibre e neiguan pode ajudar a melhorar a NVIQ em pacientes submetidos a quimioterapia.

## INTRODUCTION

Chemotherapy-induced nausea and vomiting (CINV) is the most common adverse effect in cancer patients^(1)^. Acute CINV generally occurs within minutes to hours of chemotherapy, and it can be resolved within 24 h of chemotherapy. Delayed CINV usually occurs between 2 and 5 days after chemotherapy^(2)^. The incidence of acute and delayed CINV was 55.3% and 62.3%, respectively^(3)^. CINV can cause appetite loss in mild cases, and cause malnutrition, electrolyte, acid-base balance disorder in severe cases. It can also make patients fear of chemotherapy, affect the compliance of chemotherapy and treatment effect^(4)^.

Pharmacological and non-pharmacological approaches exist for preventing and treating CINV. The American Society of Clinical Oncology clinical practice guideline recommends using neurokinin 1 receptor antagonists, serotonin receptor antagonists, dexamethasone, and olanzapine to prevent CINV, which is caused by high emetic chemotherapy. More than 60% of patients undergoing chemotherapy have been reported to experience CINV despite the use of antiemetic medications^(5)^. The national Comprehensive Cancer Network (NCCN) guidelines^(1)^ recommend acupressure, herbal therapy, music therapy, and aromatherapy, which are often cost-effective and associated with fewer side effects.

Herbal therapy was safer and less expensive alternative therapies. Ginger is most commonly used in herbal therapy. It has a long history as a folk-remedy for nausea and gastrointestinal discomfort in many cultures. Modern scientific research also confirmed that ginger might be effective against postoperative nausea and vomiting and motion sichness^(6,7)^. In recent years, some double-blind, randomized, controlled studies^(8,9)^ have used ginger to prevent CINV, which can significantly reduce gastrointestinal adverse reactions, improve the treatment compliance and life quality of patients. At the same time, ginger induced-adverse reactions in the treatment of CINV are less, and its cost is cheap and easy to obtain, so it is easy to be accepted by patients.

The most commonly used point for acupressure is the P6 point, which is called “Neiguan” in traditional Eastern medicine and is known to be associated with nausea and vomiting. By pressing the point, the energy, which is called Qi, is believed to flow easily and reduce nausea and vomiting^(10)^. Studies^(11,12)^ have shown that acupressure, as a adjuvant alternative therapy of non-invasive, safe, convenient and easy to operate complementary, can relieve CINV and improve the comfort of patients, thereby improve the life quality of patients and completion rate of chemotherapy. At the same time, the treatment cost is relatively economical, most patients can afford it. Therefore, acupressure has great promotion value in clinical practice.

The clinical application of ginger combined with P6 acupressure on CINV has not been reported. In this study, the combination of two alternative medical therapies was used to explore its effects on CINV, function living index-emesis, benefit finding, treatment satisfaction and adverse reactions.

## METHOD

### Design

The study was carried out as a randomized controlled trial involving four parallel groups. It was conducted at the Affiliated Hospital of Xuzhou Medical University between February and September 2022.

### Participants

Patients were included in the trial if they met the following criteria: diagnosed with cancer by imaging or pathology, receiving prior chemotherapy and experiencing CINV, receiving chemotherapy regimens containing oxaliplatin; aged 18 years or older, voluntarily participating in the trial and having good compliance. The exclusion criteria were as follows: vomiting caused by digestive tract obstruction, nervous system disorder, and other reasons; receiving radiotherapy before and after chemotherapy; experiencing nausea and vomiting 24 h before chemotherapy; allergic to ginger or dietary contraindications; and suffering from infectious or mental diseases.

### Interventions

#### Recruitment Process

Chemotherapy regimens were determined by clinical unit physicians, and when patients were admitted to the hospital, the first researcher contacted the physician to learn about the patient’s chemotherapy regimen and to identify potential participants. The first researcher went to the ward to communicate with patients based on the list of potential participants, patients who met the inclusion criteria were informed about the study, gave informed consent and were recruited into the study. A second researcher randomly assigned patients to one of four groups on the basis of a computer-generated random number table. The first researcher implemented the intervention, and the third researcher assisted patients in completing the necessary forms.

Control group: Routine treatment and nursing: Based on the guidelines of the NCCN, the patients in the control group received antiemetic, stomach-protecting, anti-allergic treatment diet guidance, specialist knowledge guidance and psychological nursing related health education before chemotherapy.

Ginger group: Based on the control group, patients in ginger group took two ginger capsules (each capsule containing 250 mg ginger powder) orally 30 min before chemotherapy, twice a day ^(13)^, until the end of chemotherapy. The ginger powder was produced by Shaanxi Sciphar Natural Products Co., Ltd at Senfu Health and Wellness Industrial Park, Shangzhou District, Shangluo City, Shaanxi Province.

Acupressure group: Based on the control group, Patients in the acupressure group pressed P6 point for 10 minutes 30 min before chemotherapy once a day^(14)^, until the end of chemotherapy. The P6 point was located on the anterior surface of the forearm, approximately 3-finger widths up from the crease of the wrist between the tendons of the palmaris longus and flexor carpi radialis^(15)^. The intensity of pressing was appropriate for the patient to feel sore, numb, distending, and painful using the method of pressing and kneading^(16)^.

Joint group: Based on the control group, Patients in the joint group took ginger capsule and P6 acupressure 30 min before chemotherapy, until the end of chemotherapy.

### Data Collection

Baseline data were collected on the day of the participants were recruited the study. Data on nausea, vomiting, and retching were collected 12h before chemotherapy by patients recalling in the last 12 hours at the time of recruitment and for 5 consecutive days during chemotherapy. Adverse reaction data collected on 5 consecutive days during chemotherapy. Data on functional living index-emesis, benefit finding and treatment satisfaction were collected on the fifth day.

### Outcome Measurements and Instruments

The outcome measurements and instruments of the study included: Primary outcome: nausea, vomiting and retching and functional living index-emesis. Secondary outcomes: Benefit finding, treatment satisfaction, adverse reaction.

#### Rhodes Index of Nausea, Vomiting and Retching (R-INVR)

The R-INVR scale comprised eight items, including experience time, frequency, and severity of nausea, vomiting, and retching. The scale was scored using the Likert5-point four-point method, and the total score of CINV symptoms was obtained by adding all the scores. The higher the score, the more severe the CINV symptoms^(17)^. In this study, the data on the first day of chemotherapy were regarded as acute CINV, and the data on days 2–5 were regarded as delayed CINV.

#### Hospital Anxiety and Depression Scale (HADS)

This scale was used to assess the anxiety and depression of patients. Each scale comprised seven items, with each item rated from 0 (best) to 3(worst). Seven items each were used for assessing anxiety and depression symptoms. A total score of more than 8 indicated anxiety or depression^(18)^.

#### Benefit Finding

The scale^(19)^ had 6 dimensions, and 22 items, including acceptance (item 1–3), family relations (item 4–5), world outlook (item 6–9), personal growth (item 10–16), social relations (item 17–19) and health behavior (item 20–22). The Cronbach’s *α* was 0.95^(20)^. The scale was scored using a Likert5 grade. The total score of the questionnaire was the sum of dimension scores, with a range of 22–110. The higher the score, the stronger the sense of benefit from the disease.

#### Functional Living Index-Emesis Scale (FLIE)

This scale was a self-rating scale to evaluate the impact of CINV on the daily life function of patients, including two dimensions nausea and vomiting. Each dimension had nine items. Using the Likert7 grade score, a total score greater than108 indicated that nausea and vomiting had no effect on the quality of life of patients. The research showed that the internal reliability of the scale was 0.79 and the structural reliability was from 0.74 to 0.97^(21)^.

#### Treatment Satisfaction Scale

The self-designed treatment satisfaction evaluation table was divided into five levels by referring to the literature: level 0, very satisfied and hoping to continue to use; level 1, relatively satisfactory and can be used; level 2, satisfactory and want to get oncologist’s opinion whether to use; level 3, general and can be used or not; level 4, not satisfied and want to choose it no longer^(22)^.

### Sample Size

The sample size was calculated using PASS15 software. The sample size was calculated by comparing the sample size estimation of multiple means in the random block. Due to the interventional study design, and to maximize the probability of detecting significant findings, a statistical power of 90% was used, at a confidence level of 95%. According to the results of the pre-experiment, the formula was substituted into 34. Considering the 20% dropout rate, 43 in each group(34/0.8), a total of 172 patients were included in the four groups.

### Randomization and Allocation Concealment

The second researcher generated unique random integers using a computer-generated random number table, ranging from 1 to 172, without being sorted. Each set of numbers was randomly allocated to four groups. Patients were then enrolled by the second researcher in four groups according to the pre-established list at the time of admission to the hospital. The first and second researchers could not be blinded. It was also not possible to blind the participants, due to the nature of the study. However, the third researcher was to blind due to were unaware of the patient grouping.

### Statistical Analysis

IBM SPSS Version 26.0 software was used to analyse the data, with statistical significance set at *P* < 0.05. The continuous data with normal distribution expressed as mean ± standard deviation, and the data with non-normal distribution were expressed as median; the t-test and Fisher test were used. For baseline data, analysis of variance was used for continuous data, and chi-square test and Fisher test were used for categorical data. The scores of nausea, vomiting and retching, functional living index and benefit finding were analyzed by analysis of variance. For the results with statistical differences, multiple tests in analysis of variance were used for pairwise comparison. Since this study was a comparison of four groups patients, the results of ANOVA showed significant differences indicating differences between the four groups of patients, but it was not known which two groups of patients were different, so multiple tests of variance was conducted in this study to determine the differences between the two groups. The treatment satisfaction and the adverse reactions were analyzed by chi-square test and Fisher test.

### Ethical Considerations

The study was approved by the Ethics Committee of the affiliated Hospital of Xuzhou Medical University, under number XYFY2022-KL030-01. Signed informed consent was also obtained from all patients. Chinese Clinical Trial Registry: ChiCTR2200063750.

## RESULTS

**Figure 1:**
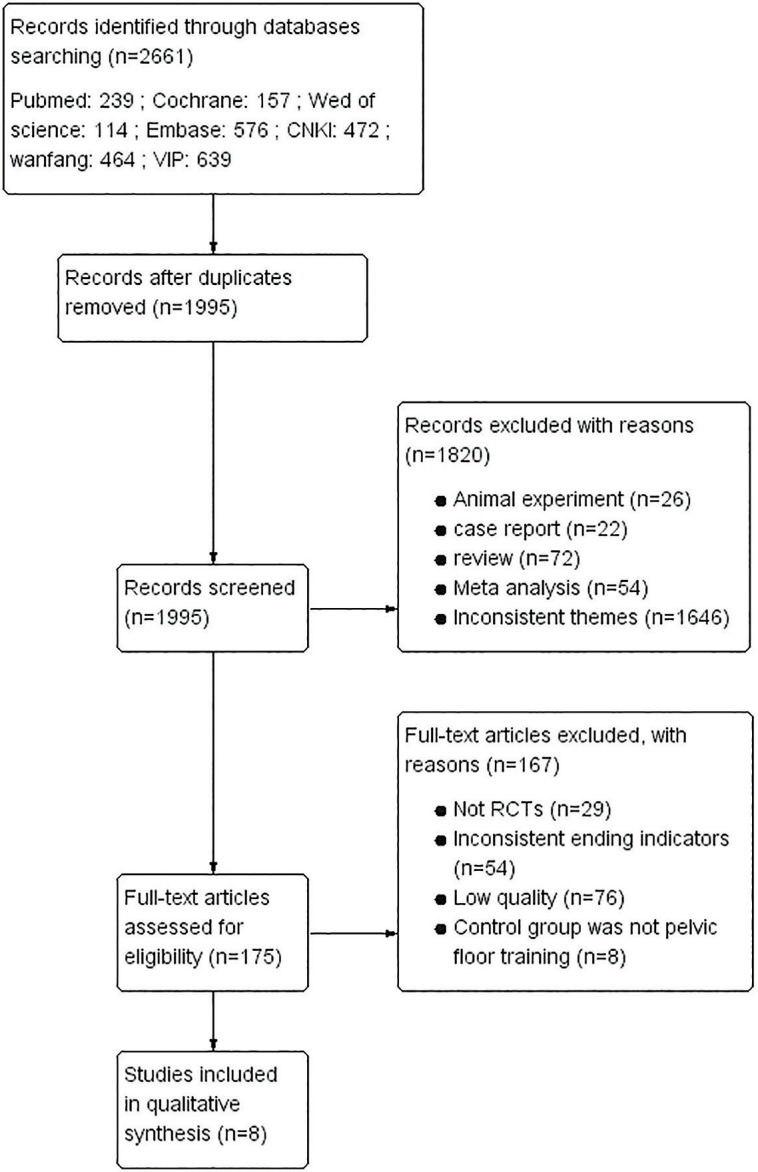
CONSORT flow diagram.

In this study, a total of 172 cancer patients were selected according to the inclusion and exclusion criteria. 3 patients in the control group withdrew from the study because of loss of follow-up, 3 patients in the ginger group withdrew from the study because they could not tolerate nausea and vomiting, and 3 patients in each of the acupoint group and the combination group withdrew from the study because of the interruption of intervention during the epidemic. In summary, a total of 160 patients were included in this study, with 40 patients in each group. The CONSORT flow diagram of the study is shown in Figure 1.

### Study Sample and Baseline Characteristics

No statistically significant difference was found among the four groups in terms of baseline characteristics (*P* > 0.05) (Table 1).

**Table 1:** Baseline characteristics among the four groups (N = 160) – Xuzhou, China, 2022.

Items		Control group	Ginger group	Acupressure group	Joint group	F/***X*** ^2^ (p)	P(p)
Age ( $\bar{\text{x}}$ ± s)		59.03 ± 1.59	57.35 ± 2.09	56.43 ± 1.60	59.55 ± 1.64	0.698^a^	0.555
Height ( $\bar{\text{x}}$ ± s)		163.28 ± 1.10	163.73 ± 1.28	164.25 ± 1.27	163.03 ± 1.17	0.199^a^	0.897
Weight ( $\bar{\text{x}}$ ± s)		59.50 ± 1.68	63.45 ± 1.73	61.50 ± 1.66	59.70 ± 1.54	1.238^a^	0.298
Body surface area ( $\bar{\text{x}}$ ± s)		1.64 ± 0.027	1.69 ± 0.027	1.67 ± 0.03	1.64 ± 0.02	0.873^a^	0.457
KPS ( $\bar{\text{x}}$ ± s)		89.75 ± 0.25	90.00 ± 0.00	89.63 ± 0.25	89.75 ± 0.25	0.491^a^	0.689
Chemotherapy cycle ( $\bar{\text{x}}$ ± s)		4.22 ± 0.36	5.08 ± 0.56	5.38 ± 0.73	5.45 ± 0.69	0.866^a^	0.460
Sex (n, %)	Male	17 (42.5%)	20 (50%)	20 (50%)	21 (52.5%)	0.901^b^	0.825
	Female	23 (57.5%)	20 (50%)	20 (50%)	19 (47.5%)		
Religion (n, %)	Yes	6 (15%)	9 (22.5%)	7 (17.5%)	4 (10%)	2.388^b^	0.496
	No	34 (85%)	31 (77.5%)	33 (82.5%)	36 (90%)		
Smoking history (n, %)	Yes	12 (30%)	13 (32.5%)	10 (25%)	14 (35%)	1.030^b^	0.794
	No	28 (70%)	27 (67.5%)	30 (75%)	26 (65%)		
Drinking history (n, %)	Yes	14 (35%)	20 (50%)	15 (37.5%)	22 (55%)	4.532^b^	0.209
	No	26 (65%)	20 (50%)	25 (62.5%)	18 (45%)		
Motion history (n, %)	Yes	18 (45%)	12 (30%)	9 (22.5%)	12 (30%)	4.922^b^	0.178
	No	22 (55%)	28 (70%)	31 (77.5%)	28 (70%)		
Sleep quality (n, %)	Well	12 (30%)	8 (20%)	12 (30%)	9 (22.5%)	2.391^b^	0.880
	General	17 (42.5%)	21 (52.5%)	19 (47.5%)	22 (55%)		
	Bad	11 (27.5%)	11 (27.5%)	9 (22.5%)	9 (22.5%)		
Difficulty concentrating (n, %)	Yes	11 (27.5%)	11 (27.5%)	10 (25%)	9 (22.5%)	0.361^b^	0.948
	No	29 (72.5%)	29 (72.5%)	30 (75%)	31 (77.5%)		
Underlying diseases (n, %)	Yes	7 (17.5%)	12 (30%)	6 (15%)	9 (22.5%)	3.137^b^	0.371
	No	33 (82.5%)	28 (70%)	34 (85%)	31 (77.5%)		
Anxiety before chemotherapy (n, %)	No	39 (97.5%)	34 (85%)	36 (90%)	35 (87.5%)	3.889^c^	0.274
	Yes	1 (2.5%)	6 (15%)	4 (10%)	5 (12.5%)		
Depression before chemotherapy (n, %)	No	33 (82.5%)	34 (85%)	34 (85%)	38 (95%)	3.234^c^	0.357
	Yes	7 (17.5%)	6 (15%)	6 (15%)	2 (5%)		
Education (n, %)	Primary school	16 (40%)	15 (37.5%)	10 (25%)	13 (32.5%)	3.162^b^	0.788
	Junior middle school	14 (35%)	17 (42.5%)	20 (50%)	16 (40%)		
	High school	10 (25%)	8 (20%)	10 (25%)	11 (27.5%)		
Occupation (n, %)	Peasant	19 (47.5%)	21 (52.5%)	20 (50%)	23 (57.5%)	3.539^b^	0.739
	Serving officer	12 (30%)	13 (32.5%)	16 (40%)	11 (27.5%)		
	Retiree	9 (22.5%)	6 (15%)	4 (10%)	6 (15%)		
Pregnancy-vomiting history (n, %)	Yes	14 (35%)	12 (30%)	9 (22.5%)	10 (25%)	1.372^b^	0.712
	No	9 (22.5%)	8 (20%)	11 (27.5%)	9 (22.5%)		
Pathological staging of tumor (n, %)	1/2 phase	15 (37.5%)	13 (32.5%)	17 (42.5%)	14 (35%)	4.497^b^	0.610
	3 phase	8 (20%)	15 (37.5%)	7 (17.5%)	9 (22.5%)		
	4 phase	14 (35%)	12 (30%)	15 (37.5%)	13 (32.5%)		
Oxaliplatin dose (n, %)	150mg	11 (27.5%)	12 (30%)	11 (27.5%)	16 (40%)	1.978^b^	0.577
	200mg	29 (72.5%)	28 (70%)	29 (72.5%)	24 (60%)		
Cancer types (n, %)	Gastric	14 (35%)	14 (35%)	10 (25%)	10 (25%)	9.037^b^	0.434
	Rectal	6 (15%)	14 (35%)	8 (20%)	11 (27.5%)		
	Colon	13 (32.5%)	7 (17.5%)	14 (35%)	10 (25%)		
	Other	7 (17.5%)	5 (12.5%)	8 (20%)	9 (22.5%)		

Annotation: a: variance analysis, b: chi-square test, c: Fisher exact method.

### Nausea, Vomiting and Retching Among the Four Groups

Significant differences were found in acute nausea and retching and delayed nausea, vomiting and retching (*P* < 0.05), but no significant difference were observed in acute vomiting among the four groups (*P* > 0.05) (Table 2). The symptoms with statistical differences among the four groups were compared, significant differences were found in acute nausea and retching and delayed nausea, vomiting, and retching in the acupressure and joint groups, and in delayed nausea and retching in the ginger group, compared with the control group (*P* < 0.05). Significant differences were found in acute and delayed vomiting in the acupressure group, and in acute and delayed nausea and retching in the joint group, compared with the ginger group (*P* < 0.05). No significant difference was noted in nausea, vomiting, and retching in the joint group compared with the acupressure group (*P* > 0.05) (Table 2).

**Table 2:** Nausea, vomiting and retching among the four groups (N = 160) – Xuzhou, China, 2022.

	Acute nausea	Delayed nausea	Acute vomiting	Delayed vomiting	Acute retching	Delayed retching
Control group	3.85 ± 3.47	4.83 ± 3.01	0.89 ± 2.01	0.70 ± 1.35	1.59 ± 1.87	2.58 ± 2.08
Ginger group	2.81 ± 2.94	2.69 ± 2.09	1.01 ± 1.81	0.44 ± 0.84	1.71 ± 1.89	1.54 ± 1.43
Acupressure group	2.45 ± 2.84	1.93 ± 2.39	0.64 ± 1.64	0.16 ± 0.47	0.83 ± 1.38	0.64 ± 1.14
Joint group	1.46 ± 2.08	0.93 ± 1.47	0.41 ± 1.13	0.13 ± 0.49	0.43 ± 1.04	0.25 ± 0.61
F	4.700	20.542	1.009	3.787	6.026	21.373
P	0.004	0.000	0.390	0.012	0.001	0.000
Control/ginger	0.109	0.000	0.740	0.187	0.725	0.001
Control/acupressure	0.031	0.000	0.507	0.006	0.033	0.000
Control/joint	0.000	0.000	0.208	0.004	0.001	0.000
Ginger/acupressure	0.574	0.142	0.320	0.153	0.013	0.005
Ginger/joint	0.037	0.001	0.112	0.108	0.000	0.000
Acupressure/joint	0.127	0.056	0.550	0.895	0.261	0.220

### Functional Living Index-Emesis Among the Four Groups

Significant differences were found in functional living index-emesis among the four groups (*P* < 0.05). (Table S1-S2) A pairwise comparison of the results was made. Compared with the control group, significant differences were observed in nausea, activity, cooking, eating, drinking liquid, and personal difficulties in the ginger group (*P* < 0.05). No significant difference was noted in functional living index-emesis (*P* > 0.05), but significant differences in all items of functional living index-nausea and vomiting were found in the P6 acupressure and joint groups (*P* < 0.05). Compared with the ginger group, significant differences were observed in the degree of nausea, activity, cooking, eating, drinking liquid, social contact, daily living, and personal difficulties in the acupressure group (*P*< 0.05); significant differences were found in the degree of vomiting, activity, eating, drinking liquid, social contact, daily life and personal difficulties in the acupressure group (*P* < 0.05); significant differences were noted in nausea and vomiting life function index in the joint group (*P* < 0.05). Compared with the acupressure group, no significant difference was noted in functional living index nausea-emesis in the joint group (*P*>0.05). (Table S3-S4)

### Benefit Finding Among the Four Groups

No significant difference was found benefit finding in acceptance, family relationships, world outlook, personal growth, social relations and health behavior among the four groups (*P*> 0.05) (Table 3).

**Table 3:** Benefit finding and adverse reaction among the four groups (N = 160) – Xuzhou, China, 2022.

Items (x ± SD)	Control group	Ginger group	Acupressure group	Joint group	F(p)	P(p)
Acceptance	12.43 ± 2.79	11.68 ± 2.68	11.60 ± 3.42	11.63 ± 3.51	0.647^a^	0.586
Family relationships	8.73 ± 1.58	8.63 ± 1.53	9.07 ± 1.37	8.98 ± 1.59	0.704^a^	0.551
World outlook	12.10 ± 2.91	11.03 ± 3.37	11.53 ± 3.57	11.93 ± 2.74	0.912^a^	0.437
Personal growth	25.30 ± 6.65	24.10 ± 5.90	26.10 ± 5.48	24.25 ± 6.63	0.926^a^	0.430
Social relationships	13.18 ± 2.83	12.00 ± 2.76	12.63 ± 3.20	13.00 ± 2.61	1.323^a^	0.269
Health behaviors	12.20 ± 2.14	11.60 ± 2.52	12.38 ± 2.06	12.55 ± 2.38	1.312^a^	0.273
Total	83.98 ± 12.24	79.03 ± 14.52	83.30 ± 12.58	82.33 ± 12.45	1.144^a^	0.333
Dizziness	1 (2.5%)	3 (7.5%)	3 (7.5%)	5 (12.5%)	2.883^b^	0.410
Headache	8 (20%)	5 (12.5%)	7 (17.5%)	5 (12.5%)	1.280^c^	0.734
Fever	0 (0%)	1 (2.5%)	2 (5%)	2 (5%)	2.271^b^	0.518
Abdominal pain	6 (15%)	4 (10%)	5 (12.5%)	4 (10%)	0.657^b^	0.883
Diarrhea	3 (7.5%)	3 (7.5%)	0( 0%)	5 (12.5%)	4.979^b^	0.676

Annotation: a: variance analysis, b: Fisher exact method, c: chi-square test.

### Treatment Satisfaction Among the Four Groups

A significant difference was observed in treatment satisfaction among the four groups (*P* = 0.004). Significant differences were found in treatment satisfaction in the acupressure and joint groups compared with the control group (*P* = 0.044/0.000), but no significant difference was noted in the ginger group (*P*= 0.074). No significant difference was observed in treatment satisfaction in the acupressure and ginger groups compared with the ginger group, (*P* = 0.084). No significant difference was noted in treatment satisfaction in the joint group compared with the acupressure group (*P* = 0.084).

### Adverse Reactions Among the Four Groups

No significant difference was found in the incidence of dizziness, headache, fever, abdominal pain and diarrhea among the four groups (*P* > 0.05) (Table 3).

## DISCUSSION

### Effect of Ginger and P6 Acupressure on CINV

Ginger could regulate gastrointestinal function and protect against gastric mucosal injury. The gastric mucosa in the gastrointestinal tract synthesized and released endogenous gastrinogen under the stimulating effect of ginger, thus avoiding damage to the gastric mucosa and exerting an antiemetic effect^(23)^. In this study, ginger powder was put into the capsule shell to reduce the irritation caused by ginger. The results of this study showed that ginger could effectively improve the symptom of delayed nausea and retching, this result was also supported by a study of the effect of ginger on CINV in breast cancer^(24)^. P6 point is one of the commonly used acupoints in the pericardial meridian of the hand Jueyin. The nerve impulses are transmitted along the spinal cord to the vomiting center by pressing and stimulating the receptors and afferent nerves in the corresponding area, thus inhibiting the abnormal discharge of the vomiting center and achieving the effect of reducing adverse reactions and stopping vomiting. P6 acupressure can effectively improve acute nausea and retching and delayed nausea, vomiting and retching. The results of one study also showed that P6 acupressure on the dominant hand was effective in reducing nausea and vomiting in patients undergoing chemotherapy^(11)^. Ginger and P6 acupressure effectively improved acute nausea and retching and delayed nausea, vomiting and retching, suggesting that ginger as an auxiliary therapy of P6 acupressure, could effectively improve acute nausea and retching.

### Effect of Ginger and P6 Acupressure on Benefit Finding and Functional Living Index Emesis

A series of adverse reactions produced by drugs seriously affect the life quality of patients during chemotherapy^(25)^. We can improve the psychological status of the patients and enhance benefit finding through the psychological nursing of the patients, which helps patients adapt to and accept the disease, makes them face life with a positive and optimistic attitude, and improve their life quality^(26)^. The results of this study showed that ginger and P6 acupressure could not effectively improve benefit finding of patients, it may be related to the short duration of intervention. However, this study found that ginger combined with P6 acupressure could effectively improve the functional living index nausea-emesis. Ginger alone could effectively improve the functional living index nausea. P6 acupressure alone significantly improve the functional living index nausea-emesis, indicating that ginger and P6 acupressure can effectively improve the quality of life of patients. The results of one study also showed that 6-gingerol 10mg orally twice daily increased the rate of complete response to CINV, appetite, and quality of life in patients receiving adjuvant chemotherapy with moderate to high emetics^(8)^. During chemotherapy, the increase in infusion time might affect the patient’s sympathetic nerves and make the patient anxious, resulting in a bad mood^(27)^. However, P6 acupressure could relax the patient’s body and mind and help relieve the bad emotion, so as to further improve life quality of patients.

### Effect of Ginger and P6 Acupressure on Treatment Satisfaction and Adverse Reactions

This study showed that the treatment satisfaction of the patients in the intervention group was higher than that in the control group, but the ginger group did not reach statistical significance, indicating that a combination of ginger and P6 acupressure were more feasible in improving patients’ adverse reactions and treatment satisfaction. After using chemotherapeutic drugs, the drug action and metabolism caused a series of adverse reactions, such as headache, dizziness, fever, abdominal pain and diarrhea. Previous studies^(28,29)^ have reported adverse effects associated with ginger, such as gastrointestinal reactions, fever, fatigue, and diarrhea. However, this study found no statistically significant difference in the incidence of adverse reactions among the four groups. It showed that the safety of the intervention method was high, with no additional increase in the incidence of adverse reactions, thus further increasing the satisfaction of patients.

### Limitations

Firstly, this study only selected patients from only one Grade A tertiary hospital in Xuzhou, with a relatively limited scope and low representation, which could not be generalized to other regions. Secondly, due to the limitation of research time, the sample size was small, and some selected research outcome indicators did not show statistical significance. Finally, this study only intervened and observed patients in one chemotherapy cycle, and failed to observe the long-term efficacy of the intervention. In the future, it is suggested that a larger sample size should be selected to reduce the research error, and longitudinal studies should be conducted to extend the intervention time, increase the follow-up time, and further observe the long-term effect.

## CONCLUSION

Ginger and acupressure, as a non-drug alternative therapy, have no obvious side effects, can relieve CINV, and improve functional life index and treatment satisfaction in cancer patients to a certain extent. However, we must emphasize that further design of the study is still needed, with studies involving a suitable blinded design, a larger sample, and a long period of intervention and follow-up to achieve a more reliable analysis. Therefore, this pilot study will serve as the basis for future clinical trials with more samples and statistical evidence.

## SUPPLEMENTARY MATERIAL

The following online material is available for this article:

Table S1 - Functional living index-nausea in four groups (N=160).

Table S2 - Functional living index-emesis among the four groups (N=160).

Table S3 - Pairwise comparison of the functional living index-nausea among four groups (N=160).

Table S4 - Pairwise comparison of the functional living index-emesis among the four groups (N=160).
