# Supplementary material for: Effect of ginger and P6 acupressure on chemotherapy-induced nausea and vomiting: a randomized controlled study
Source: Rev Esc Enferm USP. 2024 Mar 4;57:e20230104. doi: 10.1590/1980-220X-REEUSP-2023-0104en (PMC10911752; doi:10.1590/1980-220X-REEUSP-2023-0104en)
Supplement: Supplementary file 1 [file 1980-220X-reeusp-57-e20230104-suppl1-Table-S1.pdf]

Rev Esc Enferm USP
https://doi.org/10.1590/1980-220X-REEUSP-2023-0104en

Supplementary Material to "Effect of ginger and P6 acupressure on chemotherapy-induced nausea and vomiting: a randomized controlled study"

Table S1 - Functional living index-nausea in four groups (N=160).

Items	Control group (x ± SD)	Ginger group (x ± SD)	Acupressure group (x ± SD)	Joint group (x ± SD)	F(p)	P(p)
Nausea degree	3.75±1.43	4.78±1.72	5.68±1.46	6.23±0.92	23.629	0.000
Activity	3.85±1.51	4.75±1.89	5.75±1.50	6.20±1.22	18.394	0.000
Cooking	3.65±1.55	4.78±1.83	5.50±1.71	6.18±1.24	18.305	0.000
Eating	3.40±1.61	4.55±1.91	5.35±1.72	6.00±1.41	17.925	0.000
Drinking liquid	3.25±1.74	4.63±2.01	5.63±1.69	6.10±1.34	21.737	0.000
Social contact	4.50±1.43	5.05±1.92	6.00±1.18	6.60±0.59	18.958	0.000
Daily living	4.38±1.30	4.83±2.01	6.05±1.18	6.45±0.85	19.806	0.000
Personally difficulties	4.43±1.39	5.00±1.60	6.05±1.15	6.40±0.81	20.648	0.000
Relatives	6.18±1.32	6.33±1.47	6.70±0.65	6.95±0.22	4.543	0.004
Total score	37.38±11.63	44.68±14.84	52.70±11.00	57.10±7.91	22.638	0.000
