# Supplementary material for: Effect of ginger and P6 acupressure on chemotherapy-induced nausea and vomiting: a randomized controlled study
Source: Rev Esc Enferm USP. 2024 Mar 4;57:e20230104. doi: 10.1590/1980-220X-REEUSP-2023-0104en (PMC10911752; doi:10.1590/1980-220X-REEUSP-2023-0104en)
Supplement: Supplementary file 2 [file 1980-220X-reeusp-57-e20230104-suppl2-Table-S2.pdf]

Rev Esc Enferm USP
https://doi.org/10.1590/1980-220X-REEUSP-2023-0104en

Supplementary Material to "Effect of ginger and P6 acupressure on chemotherapy-induced nausea and vomiting: a randomized controlled study"

Table S2 - Functional living index-emesis among the four groups (N=160).

Items	Control group (x ± SD)	Ginger group (x ± SD)	Acupressure group (x ± SD)	Joint group (x ± SD)	F(p)	P(p)
Vomiting degree	5.40±2.00	5.65±1.76	6.38±1.23	6.85±0.48	7.995	0.000
Activity	5.38±2.07	5.63±1.81	6.38±1.21	6.85±0.48	7.956	0.000
Cooking	5.35±2.12	5.58±1.84	6.20±1.62	6.85±0.48	6.789	0.000
Eating	5.00±2.47	5.35±1.99	6.30±1.38	6.85±0.48	9.481	0.000
Drinking liquid	5.00±2.47	5.48±2.04	6.35±1.42	6.85±0.48	8.923	0.000
Social contact	5.60±1.81	5.68±1.86	6.58±1.01	6.90±0.30	8.610	0.000
Daily living	5.58±1.81	5.75±1.69	6.50±1.06	6.93±0.27	8.785	0.000
Personally difficulties	5.65±1.90	5.78±1.66	6.43±1.20	6.88±0.40	6.624	0.000
Relatives	6.50±1.16	6.38±1.41	6.95±0.22	7.00±0.00	4.721	0.004
Total score	49.45±17.09	51.25±15.05	58.05±9.81	61.95±3.34	8.724	0.000
