# Supplementary material for: Effect of ginger and P6 acupressure on chemotherapy-induced nausea and vomiting: a randomized controlled study
Source: Rev Esc Enferm USP. 2024 Mar 4;57:e20230104. doi: 10.1590/1980-220X-REEUSP-2023-0104en (PMC10911752; doi:10.1590/1980-220X-REEUSP-2023-0104en)
Supplement: Supplementary file 3 [file 1980-220X-reeusp-57-e20230104-suppl3-Table-S3.pdf]

Rev Esc Enferm USP
https://doi.org/10.1590/1980-220X-REEUSP-2023-0104en

Supplementary Material to "Effect of ginger and P6 acupressure on chemotherapy-induced nausea and vomiting: a randomized controlled study"

Table S3 - Pairwise comparison of the functional living index-nausea among four groups (N=160).

Items(p)	Control/ginger group	Acupressure /Control group	Control/ joint group	Acupressure/ Ginger group	Ginger/ Joint group	Acupressure/ joint group
Nausea degree	0.001	0.000	0.000	0.005	0.000	0.083
Activity	0.010	0.000	0.000	0.004	0.000	0.196
Cooking	0.002	0.000	0.000	0.044	0.000	0.061
Eating	0.002	0.000	0.000	0.034	0.000	0.084
Drinking liquid	0.000	0.000	0.000	0.010	0.000	0.216
Social contact	0.074	0.000	0.000	0.002	0.000	0.051
Daily living	0.152	0.000	0.000	0.000	0.000	0.203
Personally difficulties	0.045	0.000	0.000	0.000	0.000	0.221
Relatives	0.522	0.026	0.001	0.111	0.008	0.287
Total score	0.006	0.000	0.000	0.002	0.000	0.092
