# Supplementary material for: Effect of ginger and P6 acupressure on chemotherapy-induced nausea and vomiting: a randomized controlled study
Source: Rev Esc Enferm USP. 2024 Mar 4;57:e20230104. doi: 10.1590/1980-220X-REEUSP-2023-0104en (PMC10911752; doi:10.1590/1980-220X-REEUSP-2023-0104en)
Supplement: Supplementary file 4 [file 1980-220X-reeusp-57-e20230104-suppl4-Table-S4.pdf]

Rev Esc Enferm USP
https://doi.org/10.1590/1980-220X-REEUSP-2023-0104en

Supplementary Material to "Effect of ginger and P6 acupressure on chemotherapy-induced nausea and vomiting: a randomized controlled study"

Table S4 - Pairwise comparison of the functional living index-emesis among the four groups (N=160).

Items(p)	Control/ ginger group	Acupressure /Control group	Control/ joint group	Acupressure/ Ginger group	Ginger/ Joint group	Acupressure/ joint group
Vomiting degree	0.453	0.004	0.000	0.031	0.000	0.155
Activity	0.464	0.004	0.000	0.029	0.000	0.165
Cooking	0.540	0.022	0.000	0.090	0.001	0.078
Eating	0.372	0.001	0.000	0.016	0.000	0.161
Drinking liquid	0.232	0.001	0.000	0.028	0.001	0.208
Social contact	0.811	0.002	0.000	0.005	0.000	0.301
Daily living	0.564	0.003	0.000	0.014	0.000	0.162
Personally difficulties	0.692	0.015	0.000	0.041	0.001	0.156
Relatives	0.543	0.030	0.016	0.006	0.003	0.808
Total score	0.521	0.002	0.000	0.016	0.000	0.165
